# Invasive Pneumococcal Disease and Long-Term Mortality Rates in Adults, Alberta, Canada

**DOI:** 10.3201/eid2808.212469

**Published:** 2022-08

**Authors:** Kristen A. Versluys, Dean T. Eurich, Thomas J. Marrie, Gregory J. Tyrrell

**Affiliations:** University of Alberta, Edmonton, Alberta, Canada (K.A. Versluys, D.T. Eurich, G.T. Tyrrell);; Dalhousie University, Halifax, Nova Scotia, Canada (T.J. Marrie);; Provincial Laboratory for Public Health, Edmonton (G.T. Tyrrell)

**Keywords:** invasive pneumococcal disease, pneumococci, bacteria, respiratory infections, long-term mortality rates, proportional hazards model, Alberta, Canada

## Abstract

The relationship between increased short-term mortality rates after invasive pneumococcal disease (IPD) has been frequently studied. However, the relationship between IPD and long-term mortality rates is unknown. IPD patients in Alberta, Canada, had clinical data collected that were linked to administrative databases. We used Cox proportional hazards modeling, and the primary outcome was time to all-cause deaths. First IPD events were identified in 4,522 patients, who had a median follow-up of 3.2 years (interquartile range 0.8‒9.1 years). Overall all-cause mortality rates were consistently higher among cases than controls at 30 days (adjusted hazard ratio [aHR] 3.75, 95% CI 3.29–4.28), 30‒90 days (aHR 1.56, 95% CI 1.27‒1.93), and >90 days (aHR 1.43, 95% CI 1.33–1.54). IPD increases risk for short, intermediate, and long-term mortality rates regardless of age, sex, or concurrent conditions. These findings can help clinicians focus on postdischarge patient plans to limit long-term effects after acute IPD infection.

Despite introduction and recommendation of the capsular polysaccharide pneumococcal vaccine in Canada during 1989 to persons >65 years of age, *Streptococcus pneumoniae* is still a cause of major illness and death in Canada and worldwide (*1**‒**3*). The most serious manifestation of infection is invasive pneumococcal disease (IPD), which is characterized by bacteria invading normally sterile body sites, such as blood, lungs, or cerebrospinal fluid. In Canada, the incidence of IPD is around 8.8‒9.9 cases/100,000 persons (*4*,*5*) and consistently highest in persons >60 years of age (27.1 cases/100,000 men and 20.2 cases/100,000 women) (*6*).

Short-term 30-day mortality rates after IPD have been frequently studied (estimated case-fatality rate within 30 days ranging from 13% to 21%) (*7*,*8*). Increasing age and concurrent conditions are associated with increased case-fatality rates (*7*,*8*). However, studies on long-term mortality rates after IPD have been largely deficient, despite IPD being a reportable disease in Canada since 2000 (*9*).

Two studies conducted in Norway and the Netherlands investigated 1-year and 5-year mortality rates after IPD compared with those for age- and sex-matched controls in the general population (*7*,*10*). In persons who survived initial hospitalization or survived 30 days after acute infection, IPD mortality rates were higher for cases than for controls (1-year mortality rate 10%–30% for cases vs. 1%–3% for controls; 5-year mortality rate 35%–42% for cases vs. 7%–15% for controls) (*7*,*10*). However, the study noted that, in the Netherlands, most deaths occurred within the first 30 days (case-mortality rate 17%) (*7*). Thus, it remains unclear whether IPD increases long-term mortality rates. Moreover, these were highly selected samples because both studies used data from only 1 hospital in a large urban center, which are unlikely to be representative of the broader IPD population (*7*,*10*).

Widespread pneumococcal vaccination has seen major success (*11*). However, with an aging population at risk for IPD, and pneumococcal serotypes changing to evade current vaccinations, IPD remains a disease of public health concern (*12*). We have shown a change in serotypes and associated potential increases in severity of disease among IPD patients in Alberta, Canada (*13*). To determine how IPD is affecting mortality rates, we investigated short, intermediate, and long-term mortality outcomes for persons who had IPD compared with age- and sex-matched controls in Alberta over a 20-year period.

## Methods

### IPD Cases

Community cases of IPD were defined by laboratory-confirmed isolation of *S. pneumoniae* from a sterile site, including blood, cerebrospinal fluid (CSF), and pleural fluid (*14*). In Alberta, all IPD cases are reported to Alberta Health; thus, case ascertainment is accurate and complete. Data were collected on all adult IPD patients (>18 years of age) in Alberta during 1999–2019. The population of Alberta was estimated at 2.9 million at the start of follow-up and 4.3 million by the end of follow-up (*15*). Data for case-patients were collected by using standardized case reports. These data included demographic information, concurrent conditions, pharmacy data, laboratory results, diagnostic imaging, and vitals for the entirety of their hospital stay. Concurrent conditions for IPD patients have been described (*16*). This study was approved by the University of Alberta Health Ethics Research Board (Pro00071271) and Alberta Health Services.

### Matched Controls

We age- and sex-matched case-patients with up to 2 population controls who did not have a history of IPD. Because case-patients were hospitalized, where possible, hospital controls were preferred because both groups probably had poorer underlying health than nonhospitalized controls. Hospitalized controls were defined as being alive at the time of the index case, the same age (±1 year) and sex, and hospitalized within a ±3-month time frame as the case IPD diagnosis date. If >2 controls were identified, we randomly selected 2 controls from the pool of eligible controls for that case-patient. If no suitable hospitalized controls were available, we selected nonhospitalized age- and sex-matched controls from the Alberta general population registry. Unlike case-patients, who had extensive data collected as part of their hospital stays, controls had no specific data collected, other than administrative data.

### Linkage to Administrative Data

Using lifetime personal healthcare numbers (PHNs), we linked patients to the provincial administrative health databases. This linkage included Alberta Vital statistics to determine mortality rate (the provincial registry system that captures all migration within the province). We obtained all hospitalizations, ambulatory visits, and physician claims from the Discharge Abstract Database, the National Ambulatory Care Reporting System, the Ambulatory Care Classification System, and Physician Claim data. We used the standardized International Classification of Diseases, 9th and 10th Revisions (ICD-9 and ICD-10), for diagnostic coding preceding IPD date, hospitalization date for controls, or pseudodiagnosis date for nonhospitalized controls, for up to 5 years, to identify concurrent conditions.

### Outcome Measures

The primary outcome was time to all-cause mortality after IPD diagnosis date or pseudodiagnosis date for controls. We assessed short-term (<30 days after IPD), intermediate-term (30–90 days), and long-term (>90 days) mortality rates to determine the relationship between infection and survival. Mortality rates within 30 days are expected to be directly associated with acute IPD infection, as noted (*7*,*8*); intermediate and long-term mortality rates might not explicitly be from acute infection but rather a result of downstream, yet unknown, sequelae.

### Statistical Analysis

To describe the relationship between IPD patients and mortality rates, we performed logistic regression and survival analysis. Time zero was defined as date of IPD diagnosis, or pseudo-date for matched controls. Patients were followed up until death, censoring (person left the province) or March 31, 2019, if the person was alive at the end of the follow-up period. The maximum follow-up time possible was 20 years. If death or censoring preceded the start of the intermediate or long-term follow-up (for 30–90-day and >90-day analyses), we subsequently excluded those persons so as to observe the effects of IPD on these outcomes among persons who survived to these time periods. Completing the segmented analysis enabled a clearer picture of long-term mortality rates to be understood, after removing the shorter mortality rates from the estimates. Finally, an analysis was completed to look at overall survival over the entire potential 20 years of follow-up (i.e., 30-day, 30–90-day, and >90-day time periods were not assessed). If multiple IPD episodes occurred, only the first event was used.

We used Kaplan-Meier survival curves and log-rank tests to describe mortality rates over time, which we stratified by age and sex. We divided age categories into <45, 45–60, 60–75, and >75 years. To characterize the population, we identified all relevant diagnostic codes (ICD-9 and ICD-10 classifications) in the administrative databases, including hospitalization, ambulatory, and physicians claims before each persons’s respective diagnosis date. We also calculated the Elixhauser comorbidity index (*17*), which incorporates 31 comorbidities, each comorbidity category is dichotomous: it is either present or absent based on administrative coding. Thus, a person could have a range of no comorbidities (0) or upwards of all comorbidities identified (*18*). We also included 3 additional cardiovascular risk factors (hyperlipidemia, previous stroke, and previous ischemic heart disease), because cardiovascular disease is associated with increased risk for IPD. Scores were categorized into 2 groups, 0–1 comorbidities or >2 comorbidities, which is often used a marker of multimorbidity in health services research (*19*).

We used Cox proportional hazard modeling to compare case-patients and controls. We performed adjusted analyses by using models that had case-patient/control status, age categories, and Elixhauser comorbidity categories, but we used no specific model building strategy. We forced the Elixhauser comorbidity score into the model to ensure that differences in outcomes were not driven by differences in comorbidity. We also included age in our models to control for any residual confounding. We performed stratified analysis by using age, comorbidity category, and sex. In addition, to determine whether mortality rate trends have changed over time, we stratified IPD cases occurring >10 years ago, 5–10 years ago, and <5 years ago and measured this trend by using a linear trend test. Finally, we tested interactions between case status with age, sex, and comorbidity score. We tested a Cox proportional hazards assumption by using log‒log plots and Schoenfeld residuals. A p value <0.05 was considered significant in modeling. All analyses were performed by using Stata software version 15 (StataCorp LLC, https://www.stata.com).

In a sensitivity analysis, we excluded all IPD cases (and their controls) if no hospitalized controls were identified for the IPD case. In addition, we also excluded all IPD case-patients who had >1 IPD event to ensure these events were not influencing our mortality rate estimates.

## Results

### Patient Characteristics

**Figure 1:**
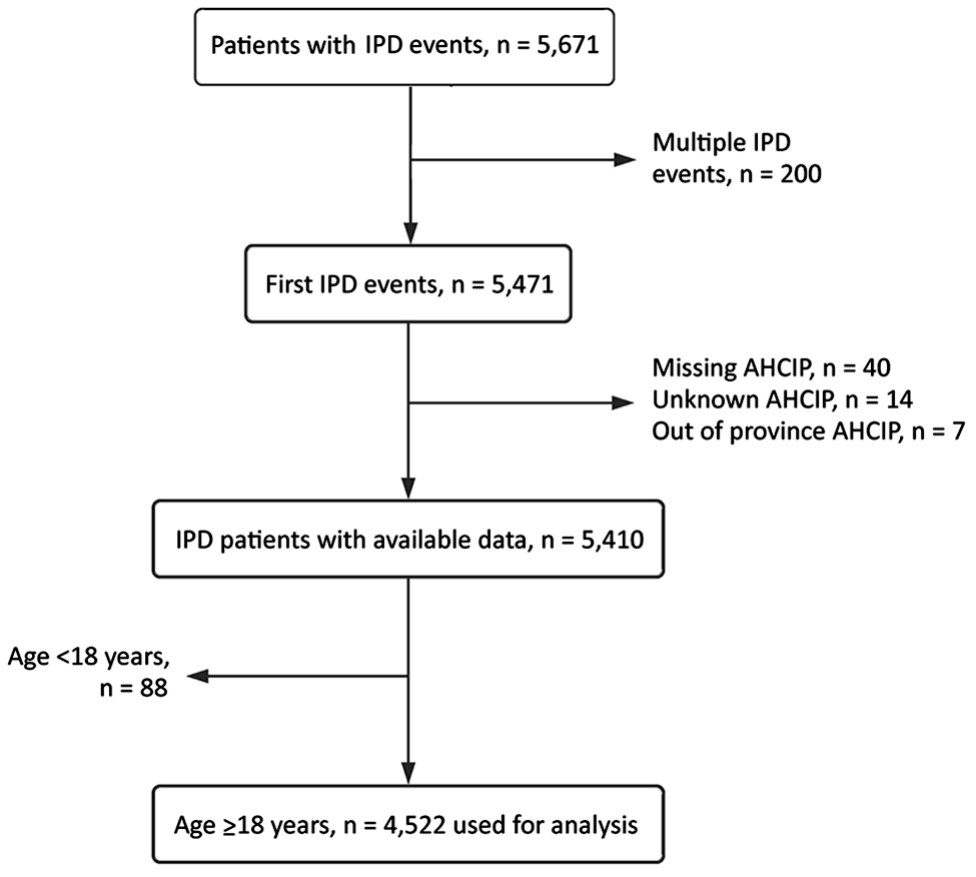
Flowchart diagram of case inclusion for study of IPD long-term mortality rates in adults, Alberta, Canada. AHCIP, Alberta Health Care Insurance Plan; IPD, invasive pneumococcal disease.

Our study comprised 4,522 IPD case-patients, and 4,315 (95%) were matched with 8,837 controls; there were 2 controls/case-patient (Figure 1). Overall, 4,357 (96.4%) IPD case-patients had >1 hospitalized control; for some IPD case-patients (n = 165, 3.6%), nonhospitalized controls were required. The mean (+SD) age of case-patients was 55.8 (+17.7) years, and 56.7% of case-patients were male; this distribution remained consistent over time (Table 1; Appendix Table 1).

**Table 1:** Characteristics for case-patients and controls for invasive pneumococcal disease long-term mortality rates in adults, Alberta, Canada*

Characteristic	Case-patients	Controls
Total	4,522 (100.0)	8,837 (100.0)
Sex		
M	2,565 (56.7)	4,994 (56.5)
F	1,957 (43.3)	3,843 (43.5)
Age, y		
<45	1,324 (29.3)	2,579 (29.2)
45‒60	1,388 (30.7)	2,735 (30.9)
60‒75	1,054 (23.3)	2,060 (23.3)
>75	756 (16.7)	1,463 (16.6)
Mean age, y (+SD)	55.8 (17.7)	55.8 (17.7)
Type of IPD		
Pneumonia	2,008 (44.4)	
Positive blood culture	1,961 (97.7)	
Positive pleural fluid	16 (0.8)	
Positive pericardial fluid	3 (0.1)	
Positive peritoneal fluid	2 (0.1)	
Unknown	26 (1.3)	
Meningitis	116 (2.6)	
Bacteremia/sepsis	2,315 (51.2)	
Unspecified type	646 (14.3)	
Unknown	1,496 (33.0)	
Median comorbidity score (IQR)	5 (2‒9)	5 (2‒8)
Comorbidities		
Asplenia	25 (0.6)	22 (0.5)
Solid organ transplant	113 (2.5)	187 (4.2)
HIV infection	136 (3.0)	41 (0.9)
Other immunosuppression conditions†	1,456 (32.2)	2,252 (50.0)
Malignancies	1,052 (23.3)	1,889 (41.9)
Chronic obstructive pulmonary disease	1,193 (26.4)	1,469 (32.6)
Other respiratory diseases‡	771 (17.1)	1,139 (25.3)
Asthma	1,045 (23.1)	1,506 (33.4)
Chronic renal disease	502 (11.1)	881 (19.6)
Hypertension	1,790 (39.6)	2,744 (60.9)
Ischemic heart disease	990 (21.9)	1,925 (42.7)
Arrhythmias	1,662 (36.8)	2,785 (61.8)
Valvular heart disease	244 (54.0)	512 (11.4)
Congestive heart failure	645 (14.3)	1,207 (26.8)
Chronic liver disease	806 (17.8)	863 (19.2)
Diabetes	1,057 (23.4)	1,768 (39.2)
Smoking	907 (20.1)	1,136 (25.2)
Harmful alcohol use§	1,968 (43.5)	2,503 (55.6)

*Values are no. (%) except as indicated. †Neutropenia, leukopenia, leukocyte disease, functional and genetic leukocyte cell abnormalities, myelofibrosis, disorders of immune mechanism. ‡Acute bronchitis, pneumoconiosis, pneumonitis, pulmonary embolism, and other pulmonary circulation disorders. §Alcohol related disorders, toxic effects of alcohol, alcoholic polyneuropathy, alcoholic cardiomyopathy, alcoholic gastritis, alcoholic fatty liver disease, alcoholic cirrhosis, alcoholic liver disease, alcohol abuse counseling and surveillance.

Data on site of infection were available for patients only during 1999–2014. Of those patients, 67% had *S. pneumoniae* identified in >1 sterile body site. There were 2,008 (44.4%) cases of invasive pneumonia, of which 1,961 (97.7%) also had a positive blood culture; the remaining 2.3% had *S. pneumoniae* isolated from another sterile body site (pleural fluid, pericardial fluid, and peritoneal fluid). There were 116 (2.6%) cases of meningitis, 2,315 (51.2%) cases of bacteremia/sepsis, and 646 (14.3%) cases from an unspecified/other sterile source (not mutually exclusive) (Table 1).

### All-Cause Mortality Rate within 30 Days

**Figure 2:**
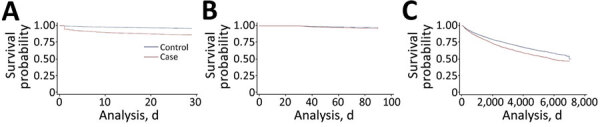
Invasive pneumococcal disease long-term mortality rates in adults, Alberta, Canada. Overall Kaplan-Meier survival estimates comparing case-patients with population controls. A) <30-day survival estimates; B) 30‒90-day survival estimates. C) >90-day survival estimates.

Within 30 days of the IPD diagnosis date (or pseudodate for controls), there were 614 deaths among IPD cases (1,915 deaths/1,000 person-years), compared with 348 deaths in the control group (510 deaths/1,000 person-years) (Figure 2, panel A). After adjustment, IPD cases were strongly associated with increased risk for 30-day mortality rate (adjusted odds ratio [aOR] 4.08, 95% CI 3.54–4.69; adjusted hazard ratio [aHR] 3.75, 95% CI 3.29‒4.28) compared with controls (Table 2; Appendix Table 2).

**Table 2:** Death outcomes of invasive pneumococcal disease (IPD) patients compared with age- and sex-matched controls, Alberta, Canada*

Time, days	Controls			Case-patients					log-rank test value, p value		
	No. positive/no. tested (%)	No. events/ 1,000 PYs		No. positive/no. tested (%)	No. events/ 1,000 PYs						
							OR (95% CI), p value			HR (95% CI), p value	
							Unadjusted	Adjusted		Unadjusted	Adjusted
<30	348/8,837 (3.9)	510		614/4,522 (13.6)	1,915		3.83 (3.34–4.39), <0.001	4.08 (3.54–4.69), <0.001	431.40, <0.001	3.65 (3.20‒4.17), <0.001	3.75 (3.29‒4.28), <0.001
30–90	220/8,459 (2.6)	107		149/3,888 (3.8)	158		1.49 (1.21–1.84), <0.001	1.58 (1.28–1.96), <0.001	14.09, <0.001	1.49 (1.21‒1.83), <0.001	1.56 (1.27‒1.93), <0.001
>90	2,086/8,236 (25.3)	37		1,174/3,733 (31.4)	49		1.35 (1.24–1.47), <0.001	1.49 (1.36–1.64), <0.001	57.44, <0.001	1.32 (1.23‒1.42), <0.001	1.43 (1.33‒1.54) <0.001
Overall	2,654/8,837 (30.0)	47		1,937/4,522 (42.8)	81		1.75 (1.62–1.88), <0.001	1.97 (1.81–2.14), <0.001	291.34, <0.001	1.66 (1.56‒1.76), <0.001	1.77 (1.67‒1.88), <0.001

*HR, hazard ratio; OR, odds ratio, PY, person-year.

We observed a major increase in risk for mortality rate in IPD case-patients in every age category for both male and female patients, as well as level of Elixhauser comorbidity category (Table 3). No interactions with age categories or Elixhauser scores were noted.

**Table 3:** Stratified hazard ratios for case-patients versus controls for invasive pneumococcal disease long-term mortality rates in adults, Alberta, Canada

Characteristic	Hazard ratio (95% CI), p value			
	<30 d	30–90 d	>90 d	Overall
Age <45 y	8.88 (5.46‒14.42), <0.001	1.75 (0.88‒3.47), 0.110	2.41 (1.97‒2.95), <0.001	2.97 (2.49‒3.53), <0.001
Age 45–60 y	4.66 (3.56‒6.09), <0.001	1.44 (0.94‒2.21), 0.098	1.64 (1.43‒1.89), <0.001	2.02 (1.80‒2.27), <0.001
Age 60–75 y	3.85 (3.00‒4.95), <0.001	1.36 (0.92‒2.00), 0.127	1.36 (1.19‒1.55), <0.001	1.69 (1.51‒1.89), <0.001
Age >75 y	2.54 (2.05‒3.15), <0.001	1.79 (1.28‒2.50), 0.001	1.10 (0.97‒1.26), 0.137	1.41 (1.27‒1.56), <0.001
Male	3.57 (3.00‒4.25), <0.001	1.48 (1.12‒1.94), 0.005	1.40 (1.27‒1.54), <0.001	1.71 (1.58‒1.85), <0.001
Female	4.02 (3.29‒4.91), <0.001	1.71 (1.24‒2.35), 0.001	1.50 (1.34‒1.66), <0.001	1.86 (1.71‒2.04), <0.001
Elixhauser score 0–1	6.41 (4.01‒10.24), <0.001	16.73 (5.09‒55.06), <0.001	1.72 (1.47‒2.02), <0.001	2.16 (1.87‒2.49), <0.001
Elixhauser score >2	3.55 (3.10‒4.08), <0.001	1.32 (1.06‒1.65), 0.015	1.36 (1.26‒1.48), <0.001	1.70 (1.59‒1.81), <0.001

When stratified by year of IPD occurrence, we found that mortality rate differences decreased over time. These decreases were for case-patients >10 years ago (1999‒2009) (aHR 4.66, 95% CI 3.75‒5.78), for case-patients 5–10 years ago (2009–2014) (aHR 4.07, 95% CI 3.09‒5.35), and for case-patients within the past 5 years (2014‒2019) (aHR 2.88, 95% CI 2.33‒3.56; p<0.001 for trend).

### All-Cause Mortality Rates at 30–90 Days

After removing IPD cases (and controls) who died or were censored within 30 days, we found that 3,888 case-patients (86.0%) and 8,459 (95.7%) controls remained. Compared with age- and sex-matched controls, for which 220 deaths (107 deaths/1,000 patient-years) occurred, we found that case-patients had 149 total deaths (158 deaths/1,000 patient-years) during 30‒90 days after the diagnosis date (Figure 2, panel B). Both unadjusted and adjusted models demonstrated an increased risk for death for persons who had IPD (unadjusted HR [uHR] 1.49, 95% CI 1.21‒1.83; unadjusted OR [uOR] 1.49, 95% CI 1.21–1.84; aHR 1.56, 95% CI 1.27‒1.93; aOR 1.58, 95% CI 1.28–1.96) (Table 2; Appendix Table 2).

When we stratified patients by age group, we observed an increased risk for death only among those >75 years of age. We observed a major increased risk for death for male and female patients, as well as by level of Elixhauser comorbidity category (Table 3). However, few events occurred in patients who had only 0–1 comorbidities (n = 31, 1.2%). No major interactions with age or comorbidities were noted.

Case-patients entering the study >10 years ago had higher observed mortality rates during 30–90 days after diagnosis date than did controls (aHR 2.12, 95% CI 1.55‒2.92). Case-patients identified during 5–10 years ago had an aHR of 1.40 (95% CI 0.84‒2.33), and those identified <5 years ago had an aHR 1.19 (95% CI 0.85‒1.66; p<0.001 for trend).

### All-Cause Mortality Rate after 90 Days

After removing IPD case-patients (and controls) who had an event within 90 days, we followed those who survived (and were not removed before 90 days) through March 31, 2019. At the end of this follow-up period, 1,174 case-patients (49 deaths/1,000 PYs) and 2,086 controls (37 deaths/1,000 PYs) died (Figure 2, panel C). Again, there was a major difference observed in mortality rates between case-patients and controls (uHR 1.32, 95% CI 1.23‒1.42; uOR 1.35, 95% CI 1.24–1.47; aHR 1.43, 95% CI 1.33‒1.54; aOR 1.49, 95% CI 1.36–1.64) (Table 2; Appendix Table 2).

Both groups that had 0–1 and >2 comorbidities showed higher mortality rates, as did every age group with the exception of patients >75 years of age. Again, female and male case-patients had similar increased risks when compared with controls. No interactions were noted. Cases identified >10 years ago had an aHR of 1.50 (95% CI 1.37‒1.64), cases from 5–10 years ago had an aHR of 1.41 (95% CI 1.20‒1.67), and cases from <5 years ago had an aHR of 1.26 (95% CI 1.06‒1.51) (p<0.001 for trend).

### All-Cause Overall Mortality Rates

When observing the entire follow-up period of 20 years, we found that the median follow-up period was 3.9 (interquartile range 1.3–10.2) years. Overall, 1,937 case-patients died (81 deaths/1,000 PYs) compared with 2,654 controls (47 deaths/1,000 PYs) (Figure 2). By age, event rates in cases were as follows: <45 years, 1,324 case-patients (29.3%), 88 deaths (14.3%); 45–60 years, 1,388 case-patients (30.7%), 170 deaths (27.7%); 60–75 years, 1,054 case-patients (23.3%), 173 deaths (28.2%); and >75 years, 756 case-patients (16.7%), 183 deaths (29.8%). Unadjusted and adjusted models provided similar results: uHR 1.66 (95% CI 1.56‒1.76), uOR 1.75 (95% CI 1.62–1.88), aHR 1.77 (95% CI 1.67‒1.88), and aOR 1.97 (95% CI 1.81–2.14) (Table 2; Appendix Table 2).

Models stratified by age, sex, and comorbidity category showed that case-patients had increased mortality rates when compared with controls (p<0.01) (Table 3). Interaction models were tested, and none were noted. log-minus-log plots and Schoenfeld residuals were generated, and no violations were noted.

**Figure 3:**
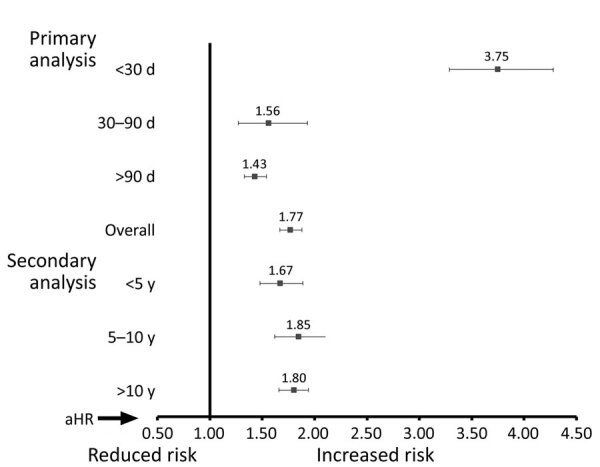
Invasive pneumococcal disease (IPD) long-term mortality rates in adults, Alberta, Canada. aHRs describing illness risk comparing IPD cases versus controls after adjusting for age and Elixhauser comorbidity scores. Primary analysis: short (<30 days), intermediate (30‒90 days), and long-term (>90 days) and overall (entire time period) follow-up. Secondary analysis: IPD cases and matched controls identified <5 years ago, 5‒10 years ago, and >10 years ago. Error bars indicate 95% CIs. aHR, adjusted hazard ratio.

Case-patients identified >10 years ago (aHR 1.80, 95% CI 1.66‒1.94]), 5–10 years ago (aHR 1.85, 95% CI 1.62‒2.11]), and in the past 5 years (aHR 1.67, 95% CI 1.48‒1.89) had similar estimates over time (p<0.01). However when compared with controls, we found that case-patients still had higher mortality rates (Figure 3; Appendix Table 3).

### Sensitivity Analysis

In a sensitivity analysis, after excluding all IPD case-patients (and their controls) if no hospitalized controls were identified for the case-patient (n = 164, 3.6%), we found that our results were unchanged (<30 days aHR 3.71, 95% CI 3.24‒4.24; 30–90 days aHR 1.43, 95% CI 1.16‒1.78; >90 days aHR 1.37, 95% CI 1.27‒1.47; overall mortality rate aHR 1.70, 95% CI 1.61‒1.81). In addition, after we excluded IPD case-patients (and their matched controls) who had >1 IPD event (n = 142, 3.1%), we observed similar results (<30 days aHR 3.92, 95% CI 3.43‒4.48; 30–90 days aHR 1.59, 95% CI 1.29‒1.97; >90 days aHR 1.39, 95% CI 1.29‒1.49; overall mortality rate aHR 1.75, 95% CI 1.65‒1.86).

## Discussion

This study showed that an episode of IPD increases the risk for death not only in the short term, which is expected, but is also a prognostic marker in the intermediate- and long-term periods. The observed aHR for 30-day mortality rate was the highest estimate because acute infection is believed to be directly associated with death. As time after infection increases, risk for death is believed to be influenced by lasting sequalae after acute infection, which this study showed remains substantial (*20*). Although the absolute difference in events per PY between cases and controls was nearly 4-fold higher in the initial 30-day period, the absolute event rate remained almost 50% higher throughout the entire follow-up period, irrespective of age or comorbidity level.

Our results were similar to those of previous studies (30-day mortality rate of 14% vs. 13%–21% published, >90-day mortality rate of 31% compared with 10%–42% previously published) (*7*,*8*,*10*,*21*). In terms of specific risk groups, like others, we observed a higher absolute rate difference in case-patients who had multimorbidities compared with those without multimorbidities irrespective of time frame (*7*,*8*,*22*). However, the relative HRs compared with those for controls were highest in persons who did not have a comorbidity. A similar trend was seen with increasing age. Although persons <45 years of age had the lowest absolute rate difference in terms of events per PY, the relative increase in deaths compared with that of controls was the highest among persons <45 years of age, and the relative difference decreased with increasing age. Although published reports frequently describe male sex as being a risk factor for increased death from IPD (*7*,*8*), our findings differ. We observed few differences in sex with respect to short- or long-term deaths. The reason for the discrepancy is unknown, but several previous studies were completed in specific populations and locations, whereas our analysis was a large population-based approach, which might partially or fully explain the reported differences. In addition, unknown confounding in previous studies or ours might also explain the differences.

Because our study spanned a wide period, it is useful to recognize advancements in medicine and preventive care for IPD. There have been decreases of aHRs over time, and the gap has decreased particularly in the past 5 years. Although the exact mechanisms of why this decrease is occurring is unknown, some possible explanations are increased use of vaccinations, herd immunity protection, and advances in use of antimicrobial drugs and supportive care (*1*). In Canada, vaccine recommendations have been consistent with the 23-valent pneumococcal polysaccharide vaccine recommended for immunocompetent adults >65 years of age (the recommended target population) and immunocompromised adults 18–65 years of age. Estimated vaccine uptake in adults >65 years of age during 2014 was ≈37% (in Canada) (*3*) and increased to ≈53% during 2020‒2021 (in Alberta) (*23*). One possible reason for the increase might be related to policy changes that enabled pharmacists in Alberta to provide routine 23-valent pneumococcal polysaccharide vaccine to eligible adults. In addition, changing serotype distribution and pathogenicity might have influenced differences in mortality rates observed between different periods.

Our study evaluated outcomes for up to 20 years in a cohort of IPD patients, covered a large sample size of persons who were identified from rural and urban areas, and had case ascertainment that is complete as a result of the provincial surveillance system and reportable requirements of IPD, but several limitations to our study should be recognized. First, because of the nature of the data, we were unable to account for some clinical differences (e.g., clinical markers such as blood pressure) that might have existed between patients who had IPD and controls. However, we adjusted for a well-known and validated Elixhauser comorbidity index, and controls were matched on site of care. Although it is not possible to adjust for every variable, our control matching on sex and age and adjustments for comorbidities provide a good understanding of IPD mortality rates. Second, the source of infection was not investigated for this study. It is hypothesized that persons who have nosocomial infections have worse outcomes than persons who have community-acquired infections (*8*), and our sample was IPD based on community-acquired infections. Third, the statistical power was low in some stratum analyses in which there were fewer deaths, in particular persons who had limited comorbidities. Thus, CIs were wide and should be interpreted with caution. Moreover, all-cause death was used as the outcome as opposed to a more cause-specific death (i.e., infectious-related death), and cause of death data were not fully available for the cohort, particularly in the early years. Fourth, history of comorbidities was based on the well-validated Elixhauser comorbidity index by using a 5-year history before diagnosis. Thus, comorbidities that might have occurred before this period for which the patient never received any subsequent care or follow-up for the condition could potentially be misclassified. Moreover, it is possible that residual confounding might exist at the level of the individual person (e.g., adherence to treatments, severity of illness) or at the population level (e.g., access to clinical care), which we could not account for in our analyses. Thus, if potential differences exist in this regard between case-patients and controls, the estimates of mortality rates could be potentially confounded. Fifth, enrollment was limited to a single province in Canada, which might limit generalizability of our findings, However, Alberta has a population of >4 million persons, so we do not see this limitation as a major concern.

In conclusion, IPD confers increased short, intermediate, and long-term mortality rates, irrespective of age or comorbidity. In particular, short-term mortality rate outcomes are most noticeable compared with those for controls. However, persons who survive past 30 days are still at increased risk for death. In aging populations at risk, combined with increasing pneumococcal serotype switching and antimicrobial drug resistance (*12*,*13*), IPD remains a major disease. Thus, focused efforts on prevention of IPD and how best to prevent downstream sequalae are required. We believe that our findings might help front-line clinicians in recognizing the high-risk nature of IPD patients, even after the acute event has been managed, and might assist in long-term postdischarge care plans and preventive strategies to mitigate the risk for longer-term adverse events in these patients.

Appendix Invasive pneumococcal disease and long-term mortality rates in adults, Alberta, Canada.
