# Supplementary material for: Invasive Pneumococcal Disease and Long-Term Mortality Rates in Adults, Alberta, Canada
Source: Emerg Infect Dis. 2022 Aug;28(8):1615–23. doi: 10.3201/eid2808.212469 (PMC9328901; doi:10.3201/eid2808.212469)
Supplement: Appendix — Invasive pneumococcal disease and long-term mortality rates in adults, Alberta, Canada. [file 21-2469-Techapp-s1.pdf]

Article DOI: https://doi.org/10.3201/eid2808.212469

# Invasive Pneumococcal Disease and Long-Term Mortality Rates in Adults, Alberta, Canada

## Appendix

**Appendix Table 1.** Demographic information comparing cases to controls, by year of IPD diagnosis or control pseudodiagnosis

| | >10 Years | | 5–10 Years | | <5 Years | |
|---|---|---|---|---|---|---|
| Characteristic | Cases | Controls | Cases | Controls | Cases | Controls |
| Totals, no. (%) | 2,014 (100%) | 3,810 (100%) | 882 (100%) | 1,758 (100%) | 1,626 (100%) | 3,269 (100%) |
| Sex, no. (%) | | | | | | |
| Male | 1,134 (56.3%) | 2,135 (56.0%) | 494 (56.0%) | 985 (56.0%) | 937 (57.6%) | 1,874 (57.3%) |
| Female | 880(43.7%) | 1,675 (44.0%) | 388 (44.0%) | 773 44.0%) | 689 (42.4%) | 1,395 (42.7%) |
| Age, y, no. (%) | | | | | | |
| <45 | 698 (34.7%) | 1,321 (34.7%) | 232 (26.3%) | 465 (26.5%) | 394 (24.2%) | 793 (24.3%) |
| 45–60 | 572 (28.3%) | 1,091 (28.6%) | 309 (35.1%) | 616 (35.0%) | 507 (31.2%) | 1,028 (31.4%) |
| 60–75 | 390 (19.4%) | 737 (19.3%) | 212 (24.0%) | 421 (23.9%) | 452 (27.8%) | 902 (27.6%) |
| ≥75 | 354 (17.6%) | 661 (17.4%) | 129 (14.6%) | 256 (14.6%) | 273 (16.8%) | 546 (16.7%) |

**Appendix Table 2.** Full model variables with adjusted hazard ratios, by time interval

| | Adjusted hazard ratios, p value (95%CI), by variable | | | | |
|---|---|---|---|---|---|
| Time, d | Case status | Age 45–60 y* | Age 60–75 y* | Age ≥75 y* | Elixhauser score ≥2† |
| <30 | 3.75, <0.001 (3.29–4.28) | 2.08, <0.001 (1.66–2.61) | 2.93, <0.001 (2.34–3.67) | 5.39, <0.001 (4.33–6.71) | 1.76, <0.001 (1.44–2.16) |
| 30–90 | 1.56, <0.001 (1.27–1.93) | 2.39, <0.001 (1.60–3.57) | 3.89, <0.001 2.62–5.75 | 7.92, <0.001 5.41–11.61 | 2.27, <0.001 (1.57–3.29) |
| >90 | 1.43, <0.001 (1.33–1.54) | 2.58, <0.001 (2.28–2.91) | 5.15, <0.001 (4.57–5.82) | 13.35, <0.001 (11.84–15.06) | 1.59, <0.001 (1.45–1.75) |
| Overall | 1.77, <0.001 (1.67–1.88) | 2.49, <0.001 (2.25–2.77) | 4.60, <0.001 (4.15–5.10) | 10.71, <0.001 (9.67–11.86) | 1.66, <0.001 (1.53–1.80) |

*Compared with age category <45 y.
†Compared with Elixhauser score <2.

**Appendix Table 3.** Adjusted hazard ratios (aHRs) for death risk, with 95% CI upper and lower limits

| Characteristic | aHR | Lower limit | Upper limit |
|---|---|---|---|
| Time from IPD diagnosis, d | | | |
| <30 | 3.75 | 3.29 | 4.28 |
| 30–90 | 1.56 | 1.27 | 1.93 |
| >90 | 1.43 | 1.33 | 1.54 |
| Overall | 1.77 | 1.67 | 1.88 |
| Time period, y | | | |
| <5 | 1.67 | 1.48 | 1.89 |
| 5–10 | 1.85 | 1.62 | 2.11 |
| >10 | 1.80 | 1.66 | 1.94 |
